# Angiotensinogen promoter methylation predicts bevacizumab treatment response of patients with recurrent glioblastoma

**DOI:** 10.1002/1878-0261.12660

**Published:** 2020-03-18

**Authors:** Thomas Urup, Linn Gillberg, Katja Kaastrup, Maya Jeje Schuang Lü, Signe Regner Michaelsen, Vibeke Andrée Larsen, Ib Jarle Christensen, Helle Broholm, Ulrik Lassen, Kirsten Grønbæk, Hans Skovgaard Poulsen

**Affiliations:** ^1^ Department of Radiation Biology Centre for Cancer and Organ Diseases Rigshospitalet Copenhagen Denmark; ^2^ Department of Oncology Centre for Cancer and Organ Diseases Rigshospitalet Copenhagen Denmark; ^3^ Department of Hematology Centre for Cancer and Organ Diseases Rigshospitalet Copenhagen Denmark; ^4^ Biotech Research and Innovation Centre (BRIC) Faculty of Health and Medical Sciences University of Copenhagen Copenhagen Denmark; ^5^ Department of Radiology Center of Diagnostic Investigation Rigshospitalet Copenhagen Denmark; ^6^ Department of Gastroenterology Hvidovre Hospital Hvidovre Denmark; ^7^ Department of Neuropathology Center of Diagnostic Investigation Rigshospitalet Copenhagen Denmark

**Keywords:** bevacizumab, DNA methylation, glioblastoma, local renin–angiotensin system, predictive biomarker

## Abstract

Patients with recurrent glioblastoma achieving response to bevacizumab combined with chemotherapy have clinical improvement and prolonged survival. High gene expression of angiotensinogen (*AGT*) is associated with a poor bevacizumab response. Because *AGT* expression is epigenetically regulated, we aimed to investigate whether *AGT* promoter methylation in tumor tissue predicts response to bevacizumab combination therapy in patients with recurrent glioblastoma. The study included 159 patients with recurrent glioblastoma, treated with bevacizumab combination treatment (training cohort, *n* = 77; validation cohort, *n* = 82). All patients could be evaluated for treatment response and biomarkers. DNA methylation of 4 CpG sites in the *AGT* promoter was measured using pyrosequencing. A model for nonresponse was established using logistic regression analysis. In the training cohort, lower methylation of each of the four CpG sites in the *AGT* promoter was significantly associated with nonresponse (all *P* < 0.05). Moreover, the mean methylation level of all four CpG sites was associated with an increased likelihood of not achieving response to bevacizumab combination therapy (twofold decrease: odds ratio = 3.01; 95% confidence interval: 1.41–6.44; *P* = 0.004). We developed a model for nonresponse in the training cohort, where a threshold of mean *AGT* promoter methylation levels was set to below 12%. The model could predict bevacizumab nonresponse with 96% specificity. Importantly, this predictor was also significantly associated with nonresponse in the validation cohort (*P* = 0.037). Taken together, our findings suggest that low *AGT* promoter methylation in tumor tissue predicts nonresponse to bevacizumab combination treatment in patients with recurrent glioblastoma. We have, thus, established and successfully validated a predictor for nonresponse that can be used to identify patients who will not benefit from bevacizumab combination therapy.

## Graphical abstract

One of four recurrent glioblastoma patients responds to bevacizumab treatment. The remaining patients have no treatment benefit. In this study, we found lower angiotensinogen (*AGT*) promoter methylation in nonresponding compared with responding patients. A predictor for nonresponse was established and successfully validated. Applying this predictor in clinical practice, one of four patients can be spared an ineffective and toxic treatment.

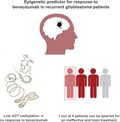

**Abbreviations**
95% CI: 95% confidence interval
AGT: angiotensinogen
AUC: area under the receiving operating characteristic curve
CEBP: CCAAT/enhancer‐binding protein
CR: complete response
EIAED: enzyme‐inducing anti‐epileptic drug
OR: odds ratio
PD: progressive disease
PR: partial response
PS: ECOG performance status
SD: stable disease
STAT3: signal transducer and activator of transcription 3
VEGF: vascular endothelial growth factor A

## 1 Introduction

Glioblastoma almost inevitable progress following standard treatment, comprising surgery, radiotherapy plus concomitant and adjuvant temozolomide (Stupp *et al.*, 2005). At tumor progression, no standard treatment is available and most known agents have shown limited activity. Bevacizumab, an antibody targeting vascular endothelial growth factor A (VEGF), has been proposed as an active drug, given that glioblastoma is characterized by an abnormal tumor vasculature fueled by pro‐angiogenic stimulation from VEGF overexpression (Baumgarten *et al.*, 2016). The inhibition of VEGF has shown to normalize the tumor vasculature and hereby improve tumor blood perfusion and drug delivery (Goel *et al.*, 2011). This mechanism of action has been confirmed in recurrent glioblastoma patients responding to anti‐angiogenic therapy on the basis of molecular and imaging data (Batchelor *et al.*, 2013; Goel *et al.*, 2011; Urup *et al.*, 2017).

Bevacizumab in combination with chemotherapy has been shown to produce high response rates of approximately 30% in recurrent glioblastoma patients (Friedman *et al.*, 2009; Urup *et al.*, 2016a; Wick *et al.*, 2017). Although this treatment has not proven active in the total population of recurrent glioblastoma patients (Taal *et al.*, 2014; Wick *et al.*, 2017), patients whom achieve response to bevacizumab combination therapy obtain clinical improvement and have prolonged survival (Henriksson *et al.*, 2011; Huang *et al.*, 2016; Jakobsen *et al.*, 2018). Especially, for the prognostic favorable group of recurrent glioblastoma patients (defined as baseline ECOG performance status ≤ 1, prednisolone ≤ 25 mg, and unifocal disease) we have based on retrospective data (Urup *et al.*, 2016a) observed an impressive difference in median overall survival (OS) of 10 months between responding and nonresponding patients. This highlights the importance of identifying patients benefitting from bevacizumab combination treatment based on prognostic factors and predictive biomarkers for bevacizumab efficacy. However, to date no validated predictive tumor biomarkers for bevacizumab efficacy have been identified.

In recurrent glioblastoma patients, a high RNA expression of the angiotensinogen gene (*AGT*) in tumor tissue has been found associated with nonresponse to bevacizumab combination therapy (Urup *et al.*, 2016b). Angiotensinogen is the primary substrate of the renin–angiotensin system, a hormone system regulating blood pressure and fluid/electrolyte homeostasis. The existence of a local renin–angiotensin system in the brain as well as in glioblastoma has been confirmed (Juillerat‐Jeanneret *et al.*, 2004; Paul *et al.*, 2006). This paracrine system regulates cerebral blood flow, and increased activity of the main effector peptide angiotensin II has been shown to reduce cerebral blood perfusion and oxygenation (Kazama *et al.*, 2004; Paul *et al.*, 2006; Vallejo‐Ardila *et al.*, 2018; Wei *et al.*, 2007). Furthermore, angiotensin II signaling has been linked to resistance to bevacizumab‐induced vascular normalization (Johansen *et al.*, 2018; Levin *et al.*, 2017; Stylianopoulos and Jain, 2013).

The transcriptional activity of *AGT* has been found to be dependent on demethylation of the CEBP (CCAAT/enhancer‐binding protein) binding site of the *AGT* promoter region (Wang *et al.*, 2014). Accordingly, the aim of this study was to investigate whether lower *AGT* promoter methylation in tumor tissue is predictive for glioblastoma patients not responding to bevacizumab combination therapy. This was initially investigated in a training cohort of 77 patients and subsequently studied in a validation cohort of 82 recurrent glioblastoma patients.

## 2 Materials and methods

### 2.1 Patients

All patients included in the study were identified using our clinical database of patients with histopathologically confirmed glioblastoma (WHO grade IV), whom at recurrence were consecutively treated with bevacizumab and irinotecan at Rigshospitalet, Copenhagen, Denmark. Eligibility criteria for the study were response evaluability and DNA methylation evaluable tumor tissue from the time of glioblastoma diagnosis. The selection of the two study cohorts is illustrated in REMARK diagrams (Figs S1 and S2).

#### 2.1.1 Training cohort

All patients treated at recurrence with bevacizumab plus irinotecan between May 2005 and December 2011 were assessed for eligibility. These patients have also been included in a previous biomarker study (Urup *et al.*, 2016b). During this period, bevacizumab (10 mg·kg^−1^) and irinotecan (125 mg·m^−2^, if EIAEDs 340 mg·m^−2^) were administered every 2 weeks and could be prescribed to recurrent glioblastoma patients in ECOG performance status 0‐2 according to published treatment protocols (Hasselbalch *et al.*, 2010; Poulsen *et al.*, 2009).

#### 2.1.2 Validation cohort

All patients treated at recurrence with bevacizumab and irinotecan between January 2012 and February 2015 were assessed for eligibility. During this period, treatment was administered according to the same protocol as for the training cohort (Poulsen *et al.*, 2009). Patients were prospectively included in the database which was regularly updated.

The study was conducted in accordance with the Helsinki Declaration, and the Danish Ethical Committee approved the study and granted exemption from the consent requirement (H‐2‐2012‐069).

### 2.2 Clinical follow‐up

Prior to administration of bevacizumab combination treatment, patients had to have measurable progressive disease (PD) by contrast‐enhanced MRI after standard therapy and be at least 4 weeks from prior chemotherapy and 3 months from completion of radiation therapy. In patients undergoing relapse surgery, bevacizumab combination therapy could be administered 4 weeks after surgery if the tumor was measurable at a postoperative MRI. Clinical follow‐up was performed every 4 weeks and MRI every 8 weeks. Treatment response was evaluated (investigator assessment by TU, VAL, and HSP) based on the Response Assessment in Neuro‐Oncology (RANO) criteria (Wen *et al.*, 2010). Patients were categorized according to their best response; patients with complete response (CR) or partial response (PR) were classified as responders, while patients with stable disease (SD) or PD were classified as nonresponders. Patients not evaluable by MRI at first response evaluation (week 8) due to early toxicity, clinical progression, or death were classified as nonevaluable and excluded.

### 2.3 Tumor sample preparation

Archived formalin‐fixed, paraffin‐embedded brain tumor tissue samples from time of initial glioblastoma diagnosis were collected, and freshly cut sections (5 μm) were stored at 2–8 °C. A pathologist (HB) performed tissue review blinded to identifiers and clinical outcome. Areas containing representative tumor cells were marked on the hematoxylin and eosin‐stained slides. In the training cohort, tumors were microdissected to enrich for tumor cells. For the validation cohort, tumors were macrodissected to increase the tumor cell frequency to above 80%.

### 2.4 DNA methylation

**Fig. 1:**
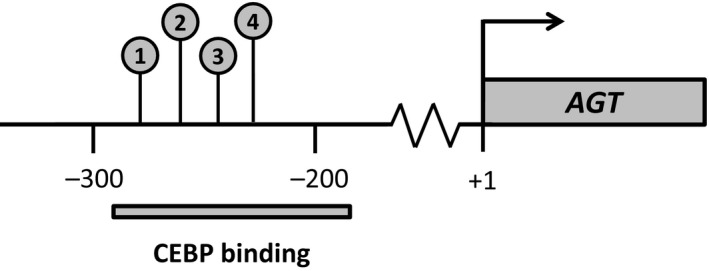
The four analyzed CpG sites in the angiotensinogen (*AGT*) promoter region. The four CpG sites analyzed by pyrosequencing were situated 282–229 base pairs (bp) upstream of the transcription start site of *AGT* in a CEBP binding region (CpG 1: −282; CpG 2: −261; CpG 3: −245; CpG 4: −229). Lower DNA methylation of these sites has been associated with a higher transcriptional activity of *AGT* (Wang *et al.*, 2014).

Genomic DNA was extracted using the QIAamp DNA Mini Kit (Qiagen, Hilden, Germany; 51304 or 51306) in the training cohort and Maxwell RSC DNA FFPE Kit (Promega; AS1450, Madison, WI, USA) in the validation cohort. DNA extracts were stored at −80 °C. DNA concentration was measured with a Qubit 2.0 Fluorometer (Thermo Fisher Scientific, Waltham, MA, USA), and genomic DNA was bisulfite‐converted using the EZ DNA Methylation‐Lightning Kit (Zymo Research, Irvine, CA, USA). DNA methylation of four selected CpG sites in the *ATG* promoter region was measured using pyrosequencing. The CpG sites analyzed were situated 282–229 base pairs upstream of the transcription start site in the *AGT* promoter (Fig. 1). The sites were selected based on a previous study where DNA methylation of these cytosines, which are situated in a CEBP binding region, has been associated with both chromatin accessibility in human adrenocortical cells and with lower *AGT* expression in both humans and rats (Wang *et al.*, 2014). Primers for PCR amplification [forward: 5′‐GGTGGTTGGTTTTAGGTTGTTATATA‐TTTA‐3′, reverse (biotinylated): 5′‐ACTATTCCCAAACTACCTATACAC‐3′] and sequencing (5′‐TGTTATATATTTA‐GGGAGATGT‐3′) were designed using the pyromark assay design 2.0 software (Qiagen). Pyrosequencing was performed using the PyroMark Q24 instrument, and pyrograms were quality‐controlled using the pyromark q24 software version 2.0.7 (Qiagen). Samples that did not pass the quality control due to low DNA amount or poor DNA quality were considered nonevaluable by methylation analysis. In the training cohort and validation cohort, five and four patients were assessed nonevaluable by methylation analysis.

### 2.5 Statistical analysis

Fisher’s exact tests and Mann–Whitney *U*‐tests were used for group comparison analyses. Survival was estimated with the Kaplan–Meier method. The probability of nonresponse was estimated by employing logistic regression, and the results are presented by odds ratios (ORs) with 95% confidence intervals (95% CIs) and the area under the receiver operating characteristic curve (AUC). Based on sample size, a fivefold cross‐validation was used for internal validation. The threshold estimated from the training set based on logistic regression analysis was used directly in the validation set in order to assess sensitivity and specificity. Continuous variables were log‐transformed (log base 2) prior to analysis. Assessment of the goodness of fit was done using the Hosmer–Lemeshow test (logistic regression). *P*‐values < 0.05 were considered significant. Calculations were performed using spss (v19.0; IBM Corp., Armonk, NY, USA), r version 3.5.0 (2018‐04‐23; R Development Core Team, Vienna, Austria, http://www.R-project.org), and sas (v9.4; SAS Institute, Cary, NC, USA).

## 3 Results

### 3.1 Patient characteristics

#### 3.1.1 Training cohort

A total of 77 recurrent glioblastoma patients were included in the training cohort. Patients were aged 23–71 years, and 62% were men. All patients had progressed after radiation therapy and temozolomide. Baseline patient characteristics and clinical outcomes are summarized in Table 1. The majority of patients (69%) were categorized into a poor prognostic group defined as having at least one of three baseline factors: poor ECOG performance status (PS = 2), corticosteroid use (prednisolone > 25 mg), or multifocal disease. After progression on bevacizumab combination treatment, 11 patients underwent surgical resection and six patients received various types of experimental treatments. At the end of follow‐up, all patients had progressed and two patients were alive (median follow‐up: 8.2 months, range: 2–69 months).

**Table 1:** Patient characteristics according to response and nonresponse to bevacizumab combination therapy in the training cohort. CI, confidence interval; CR, complete response; PR, partial response; SD, stable disease; PD, progressive disease; PFS, progression free survival; OS, overall survival.

Training cohort	Total *n = *77	Response (CR + PR) *n* = 26 (34%)	Nonresponse (SD + PD) *n* = 51 (66%)	*P*‐value
Gender, *n* (%)				
Male	48 (62)	15 (31)	33 (69)	0.62
Female	29 (38)	11 (38)	18 (62)	
Age, years (range)				
Median	56 (23–71)	54 (23–65)	57 (30–71)	0.22
ECOG performance status, *n* (%)				
0	31 (40)	12 (39)	19 (61)	0.46
1	35 (46)	12 (34)	23 (66)	
2	11 (14)	2 (18)	9 (82)	
Prior lines of chemotherapy, *n* (%)				
1	69 (90)	24 (35)	45 (65)	0.71
2	8 (10)	2 (25)	6 (75)	
Glioblastoma diagnosis, *n* (%)				
Glioblastoma	63 (82)	22 (35)	41 (65)	0.76
Secondary glioblastoma^a^	14 (18)	4 (29)	10 (71)	
Multifocal disease, *n* (%)				
Yes	21 (27)	6 (29)	15 (71)	0.60
No	56 (73)	20 (36)	36 (64)	
Corticosteroid use, *n* (%)^b^				
Yes	58 (75)	18 (31)	40 (69)	0.41
No	19 (25)	8 (42)	11 (58)	
Neurocognitive deficit, *n* (%)				
Yes	43 (56)	13 (30)	30 (70)	0.48
No	34 (44)	13 (36)	21 (62)	
Prognostic group				
Favorable^c^	24 (31)	10 (42)	14 (58)	0.44
Poor^d^	53 (69)	16 (30)	37 (70)	
Survival outcome				
Median PFS, months (95% CI)				
Total cohort	5.2	10.9 (9.6–12.3)	3.9 (3.3–4.4)	< 0.01
Median OS, months (95% CI)				
Total cohort	8.2	13.5 (10.3–16.8)	7.5 (6.3–8.6)	< 0.01
Favorable prognostic group^c^	13.3	20.3 (15.8–24.8)	8.3 (7.0–9.7)	< 0.01
Poor prognostic group^d^	7.5	8.8 (7.2–10.4)	6.5 (5.2–7.8)	< 0.01

a Lower‐grade glioma progressing as grade IV glioma.

b Prednisolone > 10 mg.

c The favorable prognostic group was defined as ECOG performance status ≤ 1, prednisolone ≤ 25 mg, and unifocal disease prior to initiation of bevacizumab combination therapy.

d The poor prognostic group was defined as having at least one of the following baseline factors: ECOG performance status = 2, prednisolone > 25 mg, or multifocal disease prior to initiation of bevacizumab combination therapy.

Twenty‐six patients (34%) achieved response to bevacizumab combination therapy. None of the clinical baseline characteristics differed significantly between the responding and nonresponding patients. Both progression‐free survival and OS were significantly longer in the responding versus nonresponding patients.

In comparison with patients belonging to a poor prognostic group presenting a median OS of 7.5 months, patients in the favorable prognostic group had a significantly better prognosis with a median OS of 13.3 months. Importantly, responding patients of the favorable prognostic group showed a better prognosis with a median OS of 20 months in comparison with 8 months in the nonresponding patients (*P* < 0.01; Table 1), resulting in a median survival difference of 12 months. In comparison, this difference in median OS was 2 months for the poor prognostic group.

#### 3.1.2 Validation cohort

Eighty‐two patients were included in the validation cohort. There were no significant differences in clinical characteristics between the validation cohort and the training cohort except for those patients in the validation cohort were older (*P* = 0.04) and had a higher frequency of multifocal disease (*P* = 0.05) (Table S1). The response rate was 33%, and clinical characteristics were not significantly associated with response (data not shown). After progression on bevacizumab combination therapy, 9 patients had surgery and 19 patients were administered various experimental treatments. At the end of follow‐up, all patients had progressed and one patient was alive (median follow‐up: 7.3 months, range: 2–40 months).

### 3.2 *AGT* promoter methylation in responding versus nonresponding patients

**Fig. 2:**
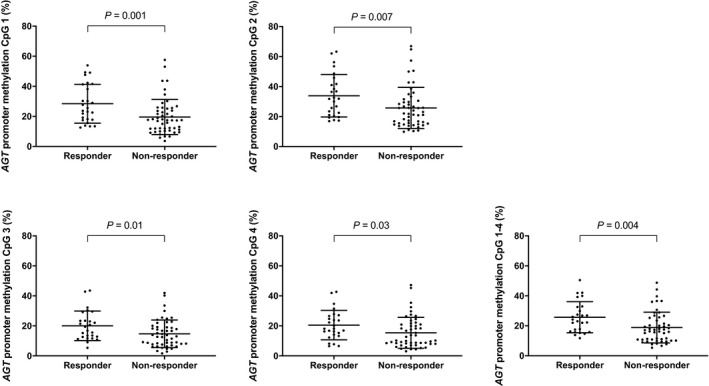
DNA methylation levels of the four analyzed CpG sites as well as the mean level of CpG sites 1–4 in the *AGT* promoter in responding and nonresponding patients of the training cohort. Mean values and standard deviations are shown by horizontal lines.

*AGT* promoter methylation levels of the four CpG sites investigated in tumor tissue from the training cohort showed a significant and high degree of intercorrelation (*P* < 0.0001 for all possible combinations; Fig. S3). *AGT* promoter methylation levels showed high variability among patients on sites 1 (range: 4–58%), 2 (range: 10–67%), 3 (range 2–44%), and 4 (range: 3–47%). The mean methylation level of the four CpG sites (mean CpG sites 1–4) ranged from 5% to 50%. As shown in Fig. 2, *AGT* promoter methylation of all four CpG sites and their mean were significantly lower in nonresponding versus responding patients (all *P* ≤ 0.03).

### 3.3 *AGT* promoter methylation as a biomarker for response

To further test whether lower *AGT* promoter methylation levels were associated with an increased likelihood of not achieving response to bevacizumab combination therapy in the training cohort, we performed univariate analyses of the candidate CpG sites. As shown in Table 2, lower methylation levels of all four CpG sites and the mean methylation of CpG sites 1–4 were significantly associated with an improved likelihood of not achieving response (all *P* ≤ 0.02). The two most significant explanatory variables were CpG site 1 and mean CpG sites 1–4 with an area under the curve (AUC) of 0.72 and 0.70, respectively.

**Table 2:** Univariate analysis of nonresponse to bevacizumab combination therapy based on *AGT* promoter methylation in the training cohort. OR, Odds ratio; CI, Confidence interval.

*AGT* promoter methylation	Nonresponse		
Twofold decrease	OR (95% CI)	*P*‐value	AUC
CpG site 1	2.93 (1.44–5.94)	0.003	0.72
CpG site 2	2.73 (1.28–5.83)	0.01	0.69
CpG site 3	2.18 (1.17–4.04)	0.01	0.67
CpG site 4	2.04 (1.14–3.66)	0.02	0.66
Mean CpG sites 1–4	3.01 (1.41–6.44)	0.004	0.70

Based on the high degree of intercorrelation of the four CpG sites and to establish the most technical robust biomarker, the mean DNA methylation level of CpG sites 1–4 was chosen, in preference to CpG site 1, for the final predictive model for nonresponse. Using this predictor, a lower mean methylation level of CpG sites 1–4 was significantly associated with nonresponse to bevacizumab combination therapy (twofold decrease: OR = 3.01; 95% CI: 1.41–6.44; *P* = 0.004).

In multivariate analysis, clinical prognostic factors (performance status, neurocognitive deficit, corticosteroid use, and multifocal disease) were not associated with response (*P *> 0.30 for all factors). However, lower mean methylation of CpG sites 1–4 was significantly and independently associated with nonresponse (*P* = 0.005).

### 3.4 Establishment of a clinical useable predictor for nonresponse

To establish a model for clinical decision making, a biomarker cut point of *AGT* promoter methylation was determined in the training cohort. Considering the difficulties and limitations in response assessment and to increase the likelihood of identifying patients not responding to bevacizumab combination therapy, we prioritized a high specificity in preference to a high sensitivity for our model. Accordingly, our predictive model with a sensitivity of 37% at a specificity of 96% was established. Based on this model, the threshold for a positive test and being considered as a ‘nonresponder’ was set to below 12% of mean *AGT* promoter methylation of CpG sites 1–4. Table 3 shows the distribution of responders and nonresponders according to the predictor. Using this predictive model, 95% (19 of 20) of the patients that were predicted nonresponders according to our model were true nonresponders. The results of the internal cross‐validation confirmed the model. In clinical practice, this means that a patient with nonmethylated *AGT* promoter (methylation < 12%) will most likely not achieve response to bevacizumab combination therapy.

**Table 3:** The predictor for nonresponse to bevacizumab combination therapy applied to the training cohort.

Training cohort	Predictor for nonresponse	
	Negative test for nonresponse Methylated (≥ 12%)	Positive test for nonresponse Nonmethylated (< 12%)
Nonresponse	32 (56%)	19 (95%)
Response	25 (44%)	1 (5%)
Total	57	20

### 3.5 Validation of the predictor for nonresponse

To validate our predictive model for not responding to bevacizumab combination treatment, DNA methylation of the four CpG sites in the *AGT* promoter was investigated in the validation cohort of similar size. Importantly, application of the predictor with the cut point of 12% methylation established in the training cohort showed that *AGT* promoter demethylation was significantly associated with nonresponse in the validation cohort (*P* = 0.037). In the training cohort, 95% (19 of 20) of the patients with nonmethylated promoter were nonresponsive (Table 3). Similarly, in the validation cohort, 88% (15 of 17) of patients with nonmethylated promoter were nonresponsive (Table 4). Considering both cohorts of the study, 23% (37 of 159) of the patients were, according to our predictive model, predicted not to respond to treatment. Of these individuals, 92% (34 of 37) patients were correctly predicted nonresponders.

**Table 4:** The predictor for nonresponse to bevacizumab combination therapy applied to the validation cohort.

Validation cohort	Predictor for nonresponse	
	Negative test for nonresponse Methylated (≥ 12%)	Positive test for nonresponse Nonmethylated (< 12%)
Nonresponse	40 (62%)	15 (88%)
Response	25 (38%)	2 (12%)
Total	65	17

## 4 Discussion

In this study of 77 recurrent glioblastoma patients, a low methylation of the CEBP binding region in the *AGT* promoter was found significantly associated with a lack of response to bevacizumab combination treatment. A clinically useful model able to predict whether a patient is likely or not to achieve response to bevacizumab combination therapy was established and validated successfully in a cohort of 82 recurrent glioblastoma patients.

Our results suggest that the methylation status of the *AGT* promoter can be used to identify recurrent glioblastoma patients, with a specificity of 96%, who will not respond to bevacizumab combination therapy. Applying this predictor in clinical practice, our data suggest that nearly one of four recurrent glioblastoma patients can be spared from an ineffective and potentially toxic treatment.

In the present study, response to bevacizumab combination treatment was found significantly associated with an improved survival. This difference in survival between responding and nonresponding patients was relatively modest for patients who were categorized as having a poor prognosis, while the difference was pronounced for patients with a favorable prognostic profile. This suggests that patients of the poor prognostic group, irrespective of response, obtain limited survival benefit of bevacizumab combination therapy.

The relatively low sensitivity of our predictive model suggests that *AGT* promoter methylation at the time of glioblastoma diagnosis is not the only factor influencing lack of response to bevacizumab combination treatment. One explanatory factor is that DNA demethylation of the CEBP region in the *AGT* promoter may occur from the time of glioblastoma diagnosis to the time of glioblastoma recurrence as a result of continues *AGT* gene activation (Wang *et al.*, 2014). Such stimulatory signals of *AGT* activation include the pro‐inflammatory cytokine interleukin‐6 and glucocorticosteroids (Wang *et al.*, 2014), which both have been reported to predict poor outcome in bevacizumab‐treated cancer patients (Duerinck *et al.*, 2015; Noonan *et al.*, 2018; Urup *et al.*, 2016a). In addition, the most prominent transcription factors known to induce angiotensinogen transcription are CEBP and STAT3 (Jain *et al.*, 2007; Wang *et al.*, 2014). In glioblastoma, these two transcriptions factors have been linked to hypoxia, necrosis, and induction of the mesenchymal subtype (Carro *et al.*, 2010; Cooper *et al.*, 2012). Consequently, DNA methylation levels of the *AGT* promoter could be reduced during tumor progression as a result of hypoxia/necrosis, inflammation, or long‐lasting corticosteroid dependency. If this is the case, angiotensinogen promoter methylation status at time of recurrence may more reliably predict treatment response. The change in *AGT* promoter methylation over time should be investigated further in longitudinal studies.

In support of our results, high protein expression of angiotensinogen in tumors from patients with metastatic colorectal cancer has been found associated with a poor response to bevacizumab combination therapy (Martin *et al.*, 2014). Accordingly, our findings may be applicable to other solid tumors. In addition, preclinical studies have shown that angiotensin II‐induced remodeling of the tumor microenvironment promotes resistance to anti‐VEGF‐induced vascular normalization (Stylianopoulos and Jain, 2013). Furthermore, increased activity of the local brain renin–angiotensin system through angiotensin II signaling has shown to cause dysregulation of cerebral blood flow by promoting cerebrovascular remodeling, vascular inflammation, and oxidative stress (Kazama *et al.*, 2004; Paul *et al.*, 2006; Wei *et al.*, 2007). Collectively, increased activity of local brain renin–angiotensin system may promote a dysregulated tumor vasculature resistant to bevacizumab‐induced vascular normalization. This could explain the findings of retrospective clinical studies, suggesting an improved benefit in terms of survival when bevacizumab is combined with an angiotensin II inhibitor (Johansen *et al.*, 2018; Levin *et al.*, 2017). Prospective clinical trials are needed to evaluate the efficacy of this combination regimen and to what extent *AGT* promoter methylation is associated with efficacy in recurrent glioblastoma patients.

This study was limited by its retrospective design, including the lack of isocitrate dehydrogenase 1 (*IDH1*) mutation status and O^6^‐methylguanine‐DNA methyltransferase (*MGMT*) gene promoter methylation status. However, *IDH1* status and *MGMT* status have previously not been associated with bevacizumab efficacy (Taal *et al.*, 2014; Wick *et al.*, 2017), suggesting that these biomarkers would not have had an impact on the results of this study.

In summary, we found promoter methylation of the gene encoding angiotensinogen as being associated with response to bevacizumab combination therapy in recurrent glioblastoma patients. Based on these findings, we established and validated a model which in clinical practice can predict patients who will not achieve response to bevacizumab combination treatment. We hypothesize that this model, in a favorable prognostic group of recurrent glioblastoma patients, has the potential to identify patients who will benefit from bevacizumab combination therapy in terms of durable response and improved survival. This hypothesis will be tested in a prospective clinical study.

## Conflict of interest

The authors declare no conflict of interest.

## Author contributions

TU, LG, UL, and HSP designed the study, and HSP coordinated it. VAL, HSP, and TU performed the RANO response evaluation. MJSL and TU analyzed the data with assistance from IJC. HB performed the histopathological evaluations. LG, KK, and KG performed the pyrosequencing analysis. TU, SRM, and LG prepared the figures and tables and wrote the manuscript with input from HSP and UL. All authors revised and approved the final version of the manuscript.

## Supporting information


**Fig. S1.** REMARK diagram for the training cohort.

**Fig. S2.** REMARK diagram for the validation cohort.

**Fig. S3.** Correlation analyses for all combinations of DNA methylation levels of the four CpG sites analyzed in the *AGT *promoter and mean CpG sites 1‐4.

**Table S1.** Patient characteristics and outcome to bevacizumab combination therapy in the training cohort and the validation cohort.
