# Supplementary figures and images for: Angiotensinogen promoter methylation predicts bevacizumab treatment response of patients with recurrent glioblastoma
Source: Mol Oncol. 2020 Mar 18;14(5):964–73. doi: 10.1002/1878-0261.12660 (PMC7191184; doi:10.1002/1878-0261.12660)

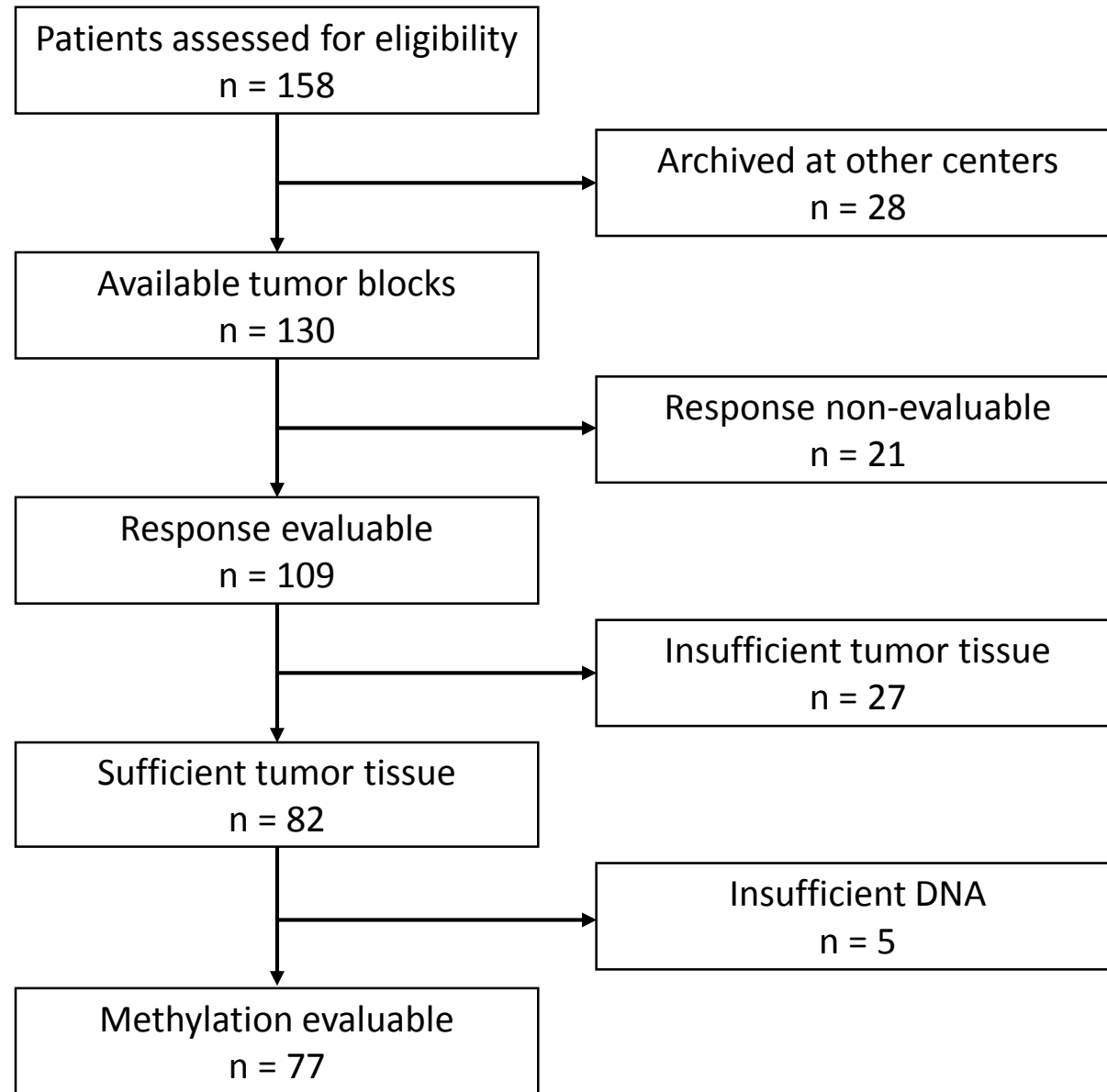

Supplement: Supplementary file 1 — Fig. S1. REMARK diagram for the training cohort. [file MOL2-14-964-s001.pdf]

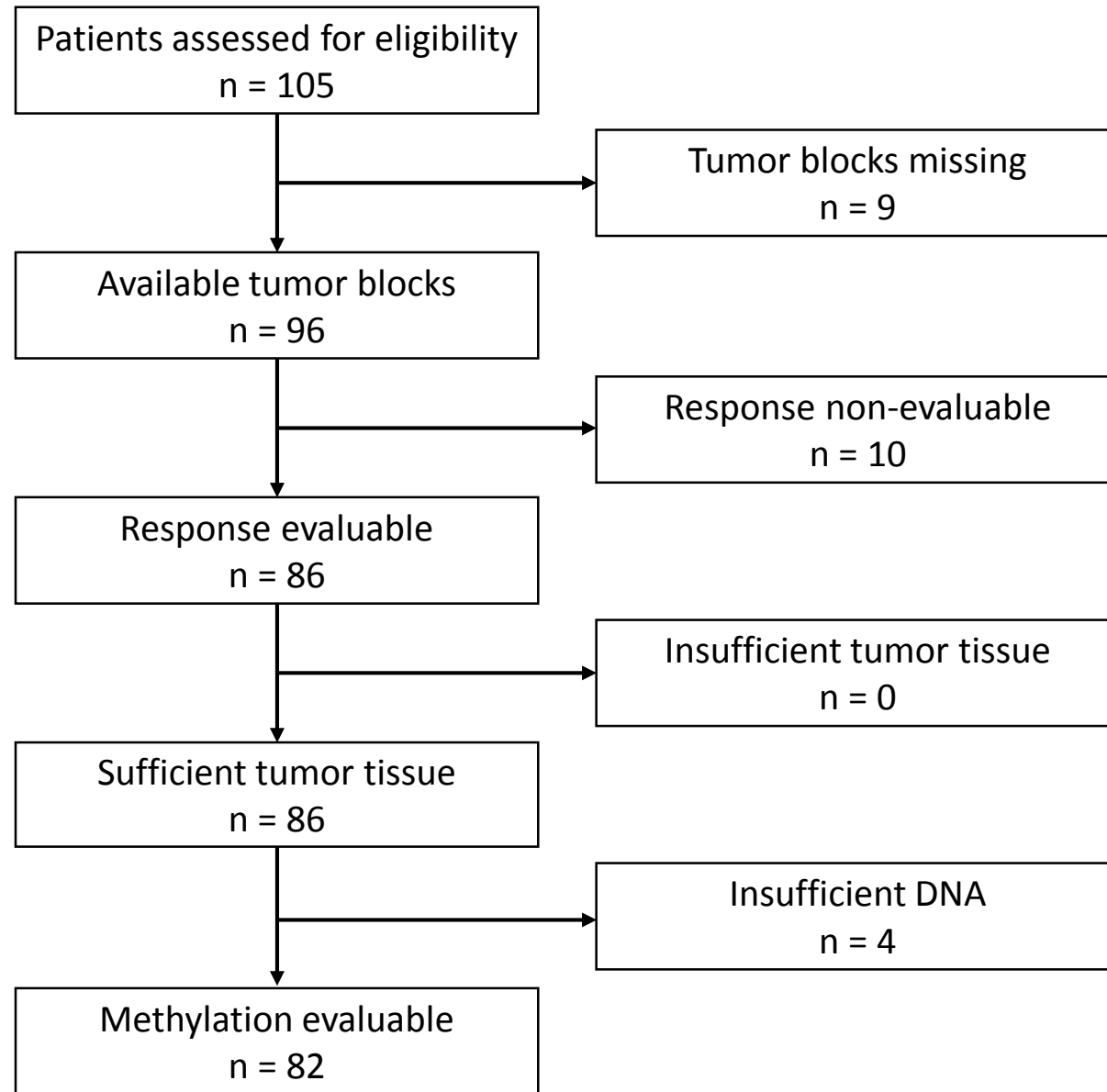

Supplement: Supplementary file 2 — Fig. S2. REMARK diagram for the validation cohort. [file MOL2-14-964-s002.pdf]

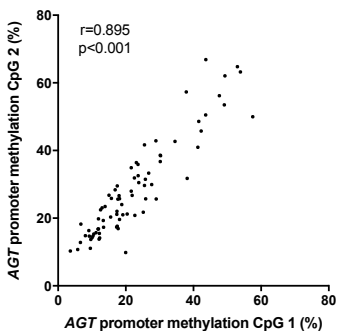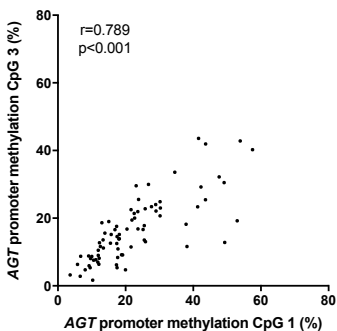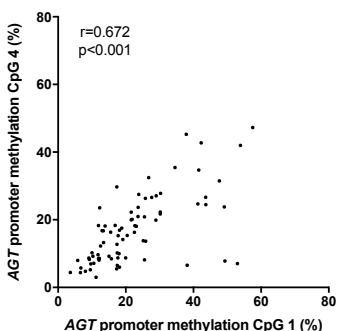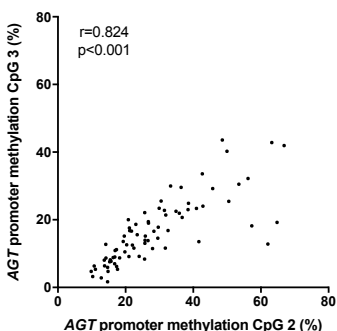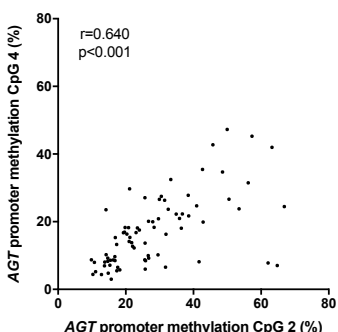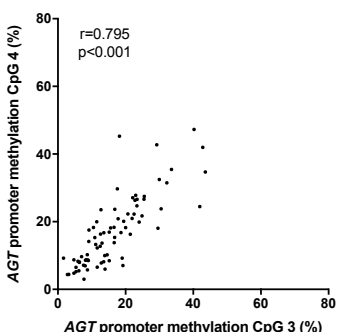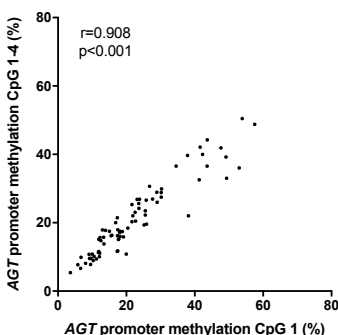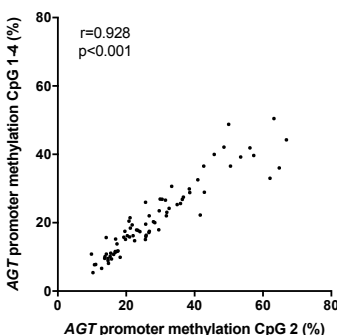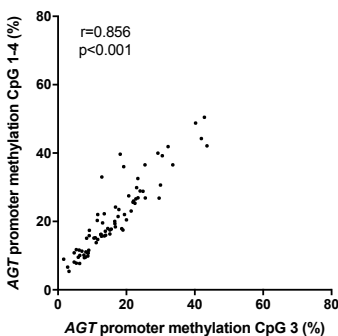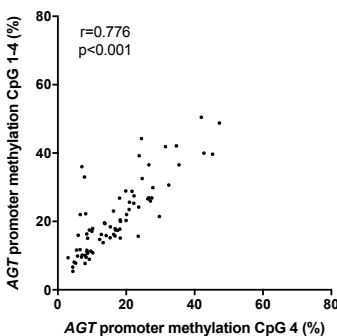

Supplement: Supplementary file 3 — Fig. S3. Correlation analyses for all combinations of DNA methylation levels of the four CpG sites analyzed in the AGT promoter and mean CpG sites 1‐4. [file MOL2-14-964-s003.pdf]
