# Supplementary material for: Angiotensinogen promoter methylation predicts bevacizumab treatment response of patients with recurrent glioblastoma
Source: Mol Oncol. 2020 Mar 18;14(5):964–73. doi: 10.1002/1878-0261.12660 (PMC7191184; doi:10.1002/1878-0261.12660)
Supplement: Supplementary file 4 — Table S1. Patient characteristics and outcome to bevacizumab combination therapy in the training cohort and the validation cohort. [file MOL2-14-964-s004.docx]

**Supplementary Table 1.** Patient characteristics and outcome to bevacizumab combination therapy in the training cohort and the validation cohort.

|  | Training cohort  (*n =* 77) | Validation cohort  (*n* = 82) | *p*-value |
| --- | --- | --- | --- |
| Gender, *n* (%) |  |  |  |
| Female | 29 (38) | 25 (31) | 0.40 |
| Male | 48 (62) | 57 (69) |  |
| Age, years |  |  |  |
| Mean (SD) | 52 (11) | 56 (11) | 0.04 |
| Median (range) | 56 (23-71) | 57 (25-79) |  |
| ECOG performance status, *n* (%) |  |  |  |
| 0 | 31 (40) | 32 (39) | 0.93 |
| 1 | 35 (46) | 36 (44) |  |
| 2 | 11 (14) | 14 (17) |  |
| Prior lines of chemotherapy, *n* (%) |  |  |  |
| 1 | 69 (90) | 69 (84) | 0.35 |
| 2 | 8 (10) | 13 (16) |  |
| Glioblastoma diagnosis, n (%) |  |  |  |
| Glioblastoma | 63 (82) | 66 (81) | 0.84 |
| Secondary glioblastoma^a^ | 14 (18) | 16 (19) |  |
| Multifocal disease, *n* (%) |  |  |  |
| Yes | 21 (27) | 35 (43) | 0.046 |
| No | 56 (73) | 47 (57) |  |
| Corticosteroid use, *n* (%)^b^ |  |  |  |
| Yes | 58 (75) | 64 (78) | 0.85 |
| No | 19 (25) | 16 (20) |  |
| Missing | 0 | 2 (2) |  |
| Neurocognitive deficit, *n* (%) |  |  |  |
| Yes | 43 (56) | 37 (45) | 0.21 |
| No | 34 (44) | 45 (55) |  |
| Prognostic group^c^ |  |  |  |
| Favourable | 24 (31) | 23 (28) | 0.73 |
| Poor | 53 (69) | 58 (72) |  |
| Response, n (%) |  |  |  |
| Response (CR+PR) | 26 (34) | 27 (33) | 1.00 |
| Nonresponse (SD+PD) | 51 (66) | 55 (67) |  |
| Median survival, months (95% CI) |  |  |  |
| Progression-free survival | 5.2 (4.0-6.3) | 4.4 (3.5-5.3) | 0.15 |
| Overall survival | 8.2 (7.3-9.1) | 7.3 (6.1-8.6) | 0.26 |
| Abbreviations: CR, complete response; PR, partial response; SD, stable disease; PD, progressive disease; 95% CI, 95% confidence interval. ^a^Lower grade glioma progressing as grade IV glioma. ^b^Prednisolone >10mg. ^c^The favourable prognostic group was defined as ECOG performance status≤1, Prednisolone≤ 25 mg and unifocal disease at baseline. | | | |
